# The Differential Characters of Measles and Scarlatina—Semiotic Value of the Expectoration in the Former

**Published:** 1846-08

**Authors:** 


*The Differential Characters of Measles and Scarlatina—Semiotic Value of the Expectoration in the former.*—Two patients affected with measles, under the care of M. Chomel, gave him an apportunity of drawing attention to the differential characters between this eruption and that of scarlatina; and of pointing out a character, peculiar to measles, by means of which, in case of doubt, the existence of the latter affection may be determined with certainty. The characters of these two eruptions, when well formed and distinct, are so well known that we need not stop to describe them; but it sometimes happens that the rash in scarlatina, instead of exhibiting a distinct, uniform redness, presents a punctuated character so similar to that of measles, that, without some degree of attention, they may readily be confounded. The following are some of the characters, by means of which this confusion may be avoided. The red points of scarlatina are equal, uniform and symmetrical. Their colour is everywhere the same, as is also the size and form of the small vesicles. In measles, on the other hand, the red points exhibit great diversity in their colour, form, and dimensions. In scarlatina there are very generally observed small miliary papulæ, which are not usually met with in measles; and fine, small subcutaneous ecchymotic spots are found in the latter, which are wanting in scarlatina. But these shades of difference, as may readily be supposed, it is not always easy to appreciate, and yet it is of importance to distinguish them, not only as regards the prognosis, but as guiding us in the selection of such prophylactic means, as we may have to prescribe in families, where one or other of these highly contagious diseases is prevalent. There is another character which we are not aware has been described by any author, but to which M. Chomel attaches the very greatest importance in a diagnostic point of view, and that is the appearance of the sputa in those affected with measles. These sputa consist of opaque nummular masses of a greyish colour, floating in a large quantity of fluid; at first sight they have all the appearance of the sputa in the second stage of phthisis; but besides the concomitant circumstances which are sufficient to guard against a mistake as to their nature and origin, the sputa in measles differ from those in phthisis in this, that whilst the fluid in which the opaque matter of the latter floats is clear, in the former it is obscure and lactescent.

This peculiar character of the expectoration, according to M. Chomel, is never absent, and though not mentioned by authors, it is perhaps because they have in general described measles as it occurs in children, and who, as is known, do not expectorate. This character, then, is of great value, in a diagnostic point of view, not only as a means of distinguishing, in doubtful cases, measles from other affections which have a great resemblance to it, but also as a means of diagnosis in those cases where the eruption has become suddenly suppressed, been imperfectly developed, or altogether wanting. It is by no means rare, especially in epidemics, to witness cases in which all the preliminary symptoms of measles may have been present, without any subsequent change manifesting itself in the skin; the slight bronchitis which almost constantly accompanies the eruption, is the only morbid phenomenon which succeeds to these initiatory symptoms. The appearance of the sputa which we have just described, will, in such cases, leave no doubt of the existence of a latent rubeola; such have been described by the ancients under the name of morbillary fever, *morbilli sine morbilis;* they might, however, with more propriety, be designated internal or bronchitic rubeola.

The following case will exhibit the value of this sign:—

A young man was admitted into the Hôtel-Dieu in such a state of stupor and oppression, that a typhoid affection was suspected. He had none of the rosy spots, however: and there was neither meteorism, nor tenderness of the abdomen. After a careful examination, an irregular violet-coloured eruption was discovered here and there upon the chest. It did not exhibit any of the appearances of the typhoid eruption, but rather resembled a morbillary eruption, which had reached its latter stage. The true nature of the eruption, however, was doubtful; but all incertitude was speedily removed, by the sputa presenting the characters we have above described. The subsequent course of the disease fully justified the correctness of the diagnosis.—*Monthly Jour. of Med. Science, from Gazette Médicale.*

